# EVALUATE THE IMPACT OF INTERDISCIPLINARY EDUCATION AND TELEHEALTH PRIMARY CARE: QUALITY AND EFFICIENCY

**DOI:** 10.1093/geroni/igad104.1435

**Published:** 2023-12-21

**Authors:** Jay Shen, Ji Yoo, Pearl Kim

**Affiliations:** School of Public Health, UNLV, Las Vegas, Nevada, United States; Kirk Kerkorian School of Medicine at UNLV, Las Vegas, Nevada, United States; School of Public Health, UNLV, Las Vegas, Nevada, United States

## Abstract

In a provider shortage area, an Interprofessional Education and Telehealth Primary Care (IPETC) for geriatrics workforce was developed in 2020. To analyze the impact of IPETC on quality and efficiency of care, we measured quality outcomes defined by the CMS defined quality measures - dementia caregiver education/referral and advance care plan, and efficiency outcomes by estimating healthcare cost savings from reducing hospitalization. Fifty eight community-dwelling adults aged 67 to 94 with mild to moderate dementia were selected in an urban safety-net primary care clinic. Propensity - demographics and level of physical impairment was matched. Main outcomes were (A) hospitalization-related healthcare cost estimates from the State Inpatient Dataset using the ICD-10 codes of principal diagnosis and hospital length of stay between January-December 2021. Twenty nine patients were cared by primary care providers who received the IPETC;29 patients were by those who did not have the IPETC; (2) CMS quality measures were compared between 2019 (baseline) and 2021. An average cost-saving of $22,135 per patient was observed among those treated by the healthcare providers who received the IPE/telehealth curriculum training than those treated by the providers without the training. CMS quality measures improved from 15.6% to 51.0% in dementia caregiver education/referral; 10.8% to 42.8% in documented advance care plans. Innovative interdisciplinary education and telehealth primary care has shown improvement of quality and efficiency of dementia care in a provider shortage area.

